# Conceptual model of pharmaceutical care for patients with coronary heart disease and comorbid conditions in Ukraine

**DOI:** 10.1080/20523211.2025.2484577

**Published:** 2025-04-08

**Authors:** Natalia Bilousova

**Affiliations:** Department of Pharmacy, Shupyk National Healthcare University of Ukraine, Kyiv, Ukraine

**Keywords:** Pharmaceutical care, cardiovascular diseases, coronary heart disease, model care, public health

## Abstract

**Background:** According to WHO’s data, a significant number of deaths from CVD is observed in Ukraine. In today’s conditions, the problems of ensuring the availability of medicines and pharmaceutical care for patients with CHD and comorbid conditions are being addressed at all levels of management of the Ukrainian healthcare sector in conditions of limited funds, resources and martial law. The scientific development of a conceptual model for providing pharmaceutical care to such patients, aimed at reducing the burden on the healthcare system and ensuring the safe use of medicines in compliance with European legislation, is highly relevant.

**Methods:** The methods used in this work include information retrieval, analysis, synthesis, generalisation, induction and deduction, synergistic, dialectical, and modelling.

**Results:** The conceptual model of pharmaceutical care for patients with CHD and comorbid conditions was developed and substantiated. The place and role of pharmacists in the context of participation in interprofessional collaboration in multidisciplinary teams is defined. The rationale for involving pharmacists in multidisciplinary teams has been substantiated, as this will enhance the quality of personalised care, prevent potential medicines interactions considering their metabolic profiles, reduce the risk of adverse reactions and improve patient adherence to pharmacotherapy.

**Conclusion:** The model of pharmaceutical care for patients with CHD and comorbid conditions takes into account the cardiovascular continuum and pharmacotherapy, which will lead to a decrease in disease progression, pharmacotherapeutic risks, reduce the burden on the healthcare system and guarantee the safety of medicines use. Implementation of the conceptual model of pharmaceutical care for patients with CHD and comorbid conditions in pharmaceutical practice requires revision of educational training programmes.

## Background

According to the Sustainable Development Goals, the World Health Organization (WHO) emphasises the development of sustainable health care systems, as stated in goal 3 (good health and well-being) (World Health Organization, n. d.). The concept of sustainable development of pharmacy emphasises the development of Global pharmaceutical services and pharmaceutical care, which ensure economic sustainability (International Pharmaceutical Federation, 2023, p. 21). Within the framework of the Global Problem of Patient Safety, attention is focused on the safe use of medicines prescribed within the scope of medical care; safety of medicines use in polypharmacy and with high cardiovascular risks patients. It is also emphasised on working in interdisciplinary teams and partnerships with patients and their families (World Health Organization, 2019). The WHO’s report raises the issue of involving pharmacists in interdisciplinary teams to guarantee the safety of medicines use within the pharmaceutical care (World Health Organization., 2019, p. 10) in order to reduce the burden on national healthcare systems.

In this regard, the International Pharmaceutical Federation (FIP) is calling on pharmacists worldwide to expand the Role of Good Pharmacy Practice (GPP) (World Health Organization, 1996, p. 10) to combat non-communicable diseases (NCDs) and implement better pharmaceutical practices. It is proposed to focus all efforts on primary prevention, screening, pharmaceutical care, therapy management of diseases such as cardiovascular (CVD), mental health, diabetes, asthma/chronic obstructive pulmonary disease, oncology, etc. Special attention is paid to the training of pharmaceutical personnel to work in general pharmacies and hospital pharmacies as part of multidisciplinary professional teams. As examples, the best pharmaceutical practices with proven effectiveness of countries such as Australia, China, Finland, Germany, India, Nigeria, Portugal, South Africa, Sweden, Switzerland and the USA are given (International Pharmaceutical Federation, 2022, p. 24). In the context of the above questions, it is proposed to provide access to pharmacists (ambulatory cards with the history of medicines intake, cloud databases of health care institutions) to the patients’ medicines profile for further analysis of prescribed drug pharmacotherapy. This information should include the names of medicines, composition, dosage, mode of medicines administration, routes of medicines administration, duration of pharmacotherapy and information about the doctor who prescribes medicines (International Pharmaceutical Federation, 2019, p. 127).

The handbook for pharmacists with CVD, developed by FIP, which offers a comprehensive approach to the specified problem, requires special attention. The document takes into account the issues of introducing pharmaceutical care into pharmaceutical practice by expanding the roles of pharmacists and reducing the burden on national healthcare systems of such measures as screening, primary and secondary prevention of modified risk factors for cardiovascular cases (dyslipidemia, diabetes), smoking, obesity and metabolic syndrome, stress, sleep disorders and psychosocial factors. The listed actions are proposed to be implemented by clinical pharmacists of healthcare facilities and general pharmacy facilities (International Pharmaceutical Federation, 2022, p. 24).

For the Ukrainian healthcare system, this problem is exacerbated as a result of the state of war, which was provoked by the russian attack. Accordingly, sociological research conducted jointly with the Kharkiv Institute of Social Research and Médicos del Mundo (Organisation ‘Doctors of the World') revealed the key barriers in the population’s access to medical care (Kharkiv Institute of Social Research, 2024, p. 25). Obstacles were the cost of pharmaceuticals, reduction of medical and pharmaceutical personnel compared to 2021 by 13.7% (89,000 people), deficit of the healthcare budget (11.4% of the total) (Ministry of Finance of Ukraine, 2024) and overloading of the healthcare system. At the same time, there is an increase in cardiovascular diseases and their complications, diabetes, post-traumatic stress disorders and panic attacks (Kharkiv Institute of Social Research, 2024, p. 25).

Therefore, the involvement of pharmacists to the interdisciplinary teams for the provision of pharmaceutical care in the structure of medical care is timely and requires the development of a conceptual model for the provision of pharmaceutical care to patients with CVD, in particular, CHD with comorbid conditions in order to reduce the burden on the healthcare system and guaranteeing the safety of medicines use (Euro Commission, 2023).

The analysis of Ukrainian scientific works in the field of providing pharmaceutical care to patients with CVD indicates the beginning of the work of domestic scientists on this problem. Thus, in scientific studies, the issues of providing pharmaceutical assistance in the field of pharmaceutical support for patients with arterial hypertension (AH) and under the ‘Affordable Medicines' programme are revealed (Malanchuk et al., 2022, pp. 26–34; Yatskova et al., 2019, pp. 31–42); monitoring of the safe use of medicines for chronic diseases in the field of pharmaceutical care (Tkachenko, Pankevych et al., 2024, p. 64); quality management of pharmaceutical care for patients with acute cerebrovascular cases in the structure of medical care in hospital pharmacy institutions (Levytska, 2020, pp. 81–88); the problems of self-treatment of the population with CVD are raised through the Google search system using the definition of common trends and keywords (Bilousova, 2024a, pp. 151–159); the influence of socio-economic factors in the conditions of the war in Ukraine on the growth of CVD and the introduction of pharmaceutical care services into pharmaceutical practice is determined (Bilousova & Mykhalchuk, 2024, pp. 347–355). However, we note that the issue of providing pharmaceutical care to patients with CVD is fragmented and requires new scientific approaches based on the principles of modern compliance with the European regulatory documents of the Ukrainian legal framework, taking into account personalised approaches to patients.

## Methods

The basis (materials) for the scientific justification and development of a conceptual model of pharmaceutical care were scientific research by foreign scientists, as well as European, American and Ukrainian regulatory and legal support for the provision of medical and pharmaceutical care to patients with coronary heart disease with comorbid conditions.

The research includes methods of information search, analysis, synthesis, generalisation, induction and deduction, synergistic, dialectical and of modelling.

To analyse the regulatory and legal documents of Ukraine regarding the provision of medical care to patients with coronary heart disease with comorbid conditions, as well as its pharmaceutical component, a search was conducted using the following keywords: ‘medical guarantees program' (59 documents), ‘cardiovascular diseases’ (4 documents), and ‘essential medicines' (28 documents). The search was performed on the website of the Verkhovna Rada of Ukraine, specifically in the ‘Legislation' section under the subsection ‘Regulatory and legal framework of Ukraine' (Parliament of Ukraine, n.d.).

Additionally, on the website of the State Expert Center of the Ministry of Health of Ukraine, within the section ‘Standardization in the field of health care' and the subsection ‘Standardization of medical care,' a review was conducted in the register of ‘Medical and technological documents' (State Expert Center of the Ministry of Health of Ukraine, n.d.). A total of 16 documents were selected based on the following topics: ‘coronary heart disease’ (3 documents), ‘arterial hypertension' (3 documents), ‘type 2 diabetes' (3 documents), ‘heart failure' (3 documents), ‘atrial fibrillation' (3 documents), and ‘pharmacist's protocol for dispensing reimbursed medicines to patients with cardiovascular diseases' (1 document). Documents that were duplicates with different dates of amendments were excluded. Only regulatory documents with the most recent update as of the time of writing this article were included in the analysis. As a result, a total of 107 regulatory documents were selected for this study.

Given that European and American clinical guidelines are utilised in Ukrainian medical practice, we reviewed the websites of the European Society of Cardiology (ESC) (ESC Guidelines & Scientific Documents, n.d.), the American Heart Association (AHA) (Guidelines and Statements, n.d.), and the American Diabetes Association (ADA) (Practice Guidelines Resources | American Diabetes Association (n.d.). Relevant clinical guidelines for the management of patients with coronary artery disease, hypertension, atrial fibrillation (AF), heart failure (HF), and type 2 diabetes were selected. A content analysis was subsequently conducted to isolate the pharmaceutical component from these clinical guidelines and related medical and technological documents. Additionally, a search was conducted for international documents regulating pharmaceutical care as part of medical care for patients with coronary heart disease and comorbid conditions. This search involved the websites of the International Pharmaceutical Federation (FIP), using the keywords ‘pharmaceutical care' and ‘cardiovascular diseases’ (2 documents) (FIP Library, n.d.); the European Society of Clinical Pharmacists (ESCP) (1 document) (Content restricted – ESCP, n.d.); and the American Pharmaceutical Association (APhA) (5 documents) (Chronic Disease Management, n.d.).

Furthermore, a narrative review of scientific sources was carried out using the keywords: ‘pharmaceutical care,' ‘pharmaceutical care model,' ‘cardiovascular diseases,' ‘coronary heart disease,' ‘chronic coronary syndrome,' ‘pharmaceutical services,’ and ‘quality indicators of pharmaceutical care' in English and Ukrainian. The search covered the past 10 years and included the scientometric databases Scopus, Web of Science, PubMed, and the Cochrane Library. A total of 9,575 scientific and informational sources were identified, out of which 237 scientific papers were content-analysed. Exclusion criteria included abstracts, duplicate publications, paid-access scientific publications, and sources indirectly related to the research topic. After analysing the regulatory documents and scientific publications, we applied the synthesis method to integrate the research findings and develop a holistic approach for formulating a conceptual model of pharmaceutical care. Using the induction method, we generalised conclusions about existing global models of pharmaceutical care. The deduction method enabled us to combine the results of the information search and logically integrate them into a framework for building a model within the Ukrainian healthcare system.

Through the synergistic method, we identified approaches to enhancing collaboration among patients, pharmaceutical professionals, medical practitioners, and the Ukrainian healthcare system. This method also revealed gaps in interprofessional interactions between participants in pharmaceutical care. The dialectical method was employed to investigate contradictions within the existing Ukrainian pharmaceutical care model. These analyses allowed for the modelling of pharmaceutical care model as a unified, continuous process, taking into account existing Ukrainian regulatory documents, identified gaps, and synergistic integration into the broader concept of providing medical care to patients with coronary artery disease and comorbid conditions.

In this study, we did not assess the sustainability of the developed pharmaceutical care model or its pharmacoeconomic impact on the Ukrainian healthcare system budget (Babar, 2024), which will be the focus of our future research.

## Results

The international documents we have analysed indicate the relevance of implementing pharmaceutical care and redirecting the activities of pharmacies towards providing pharmaceutical care services to patients (International Pharmaceutical Federation, 2019, p. 127; 2023, p. 21; Ministry of Finance of Ukraine, 2024; World Health Organization, 1996, p. 10) in addition to pharmaceutical provision within the framework of improving access to medicines.

Given the adoption of Resolution CM/Res (2020) (Euro Commission, 2023) by the European Commission and the implementation of measures within the project activities of pharmacy institutions in European countries to provide pharmaceutical care within the structure of medical care in healthcare institutions and general pharmacies, a number of issues have been identified that are relevant to medical and pharmaceutical personnel in the context of patient safety. These issues include questions regarding:

1. polypharmacy with simultaneous use of fixed combinations of medicines;
2. medicines interactions;
3. unwanted side effects;
4. commitment to the treatment of patients;
5. screening of disease risk factors;
6. prediction of pharmacotherapeutic risks (Henman et al., 2024, p. 232).

Solving these problems will help to refer patients to healthcare facilities in a timely manner, prevent the development of NCDs, and improve adherence to treatment (Henman et al., 2024, p. 232).

In Ukraine, at the state level in 2023, a new edition of the ‘Law on Medicines' (Cabinet of Ministers of Ukraine, 2022) was adopted, which for the first time introduces the wording of the term ‘pharmaceutical assistance' at the legislative level and emphasises interprofessional interaction within the multidisciplinary teams, rational and safe use of pharmaceuticals, taking into account personalised approaches to achieve better clinical results and monitoring and evaluation of pharmacotherapy (Ministry of Health of Ukraine, 2022). Also, for the first time, GPP is officially defined as an industry standard that implements state policy in the field of healthcare and defines a set of rules for the provision of pharmaceutical services and pharmaceutical care in order to implement effective pharmacotherapy (Cabinet of Ministers of Ukraine, 2022). It should be noted that previously GPPs were of a recommended nature. And the ‘Law on Medicines' officially enters into force after the end of martial law. At the industry level, updated pharmacist protocols were adopted in 2022, including the provision of pharmaceutical care to patients with CVD. These protocols are aimed at the implementation of pharmaceuticals for reimbursement and training of patients in the safe use of pharmaceuticals (Ministry of Health of Ukraine, 2022).

The analysed documents contain controversial data on the provision of pharmaceutical care to patients with CVD and require the updating of pharmacists’ protocols for the safe use of pharmaceuticals. Since the pharmacotherapy for CVD is quite diverse, contains fixed combinations of medicines and sometimes requires the simultaneous use of up to 10 medicines or even more, one should carefully approach the rational and safe use of medicines, guided by clinical recommendations based on clinical evidence of the effectiveness of medicines and best clinical practices, prejudging the occurrence of a possible interaction of medicines and adverse reactions to medicines in view of the pharmacological properties of medicines and their metabolic profile.

Separately we should note that in medical practice, clinical recommendations, guidelines and protocols for providing medical care to patients with CHD with comorbid conditions are used (Kovalenko et al., 2023, p. 84; Ministry of Health of Ukraine, 2021a; 2021b), which are regularly revised in accordance with the updates of the clinical recommendations of the European Society of Cardiology (ESC).

Attention is drawn to the clinical recommendations of the American Heart Association (AHA) for the management of patients with chronic coronary syndrome with proven pharmacoeconomic efficiency, in which the involvement of pharmacists within the interprofessional interaction in multidisciplinary teams is recommended. Pharmacists are suggested to monitor and manage the effectiveness of CHD pharmacotherapy within the framework of providing pharmaceutical care, as well as to monitor possible side effects and medicines interactions (Virani et al., 2023) Accordingly, the American Pharmaceutical Association (APhA) recommends that pharmacists use the clinical recommendations proposed by the AHA, which include recommendations for patients with dyslipidemia, arterial hypertension, myocardial infarction, thromboembolism, atrial fibrillation and heart failure (APhA., n.d.). Therefore, the provision of pharmaceutical care for CHD in the USA is built on the basis of the cardiovascular continuum and enables intervention and prevention of the development of cardiovascular cases and premature death of patients (Dzau & Braunwald, 1991, pp. 1244–1263; Zamorano JL., 2008, pp. 17–21).

In the European clinical guidelines for the treatment of patients with CHD with accompanying diabetes, the ESC also recommends the involvement of pharmacists within the interprofessional interaction as part of multidisciplinary teams (Marx et al., 2023). It should be noted that the implementation of guidelines for the prevention of CVD is evaluated using observational studies of European actions on primary and secondary prevention through interventions and with the aim of reducing cardiovascular cases (EUROASPIRE IV, V) (De Smedt et al., 2019; Kotseva et al., 2020).

Also, we should note that the effectiveness of the intervention of pharmacists in providing pharmaceutical care on adherence to treatment of the patients with CHD was proven in the randomised clinical trial (RCT) MIMeRIC (n = 316), conducted by Swedish scientists M. J. Östbring, T. Eriksson, G. Petersson, L. Hellström (Östbring et al., 2021, p. 367). The results of a systematic review and meta-analysis of RCTs (n = 8933 patients with CVD) conducted by a group of researchers from Great Britain A. A. Alshehri, Z. Jalal, E. Cheema and others testify to the improvement of clinical data on blood pressure, glycated hemoglobin, low-density lipoproteins, as well as adherence to pharmacotherapy of patients with CVD in the context of primary prevention and control of modified risk factors under the guidance of pharmacists (Alshehri et al., 2020, pp. 29–38).

The pharmacoeconomic efficiency of prescribing pharmacotherapy to patients with hypertension by pharmacy pharmacists (n = 121) was proven by American researchers D. L. Dixon, K. Johnston, J. Patterson, and others. Their modelled and conducted cost-effectiveness analysis of the involvement of pharmacists in the treatment of hypertension proved savings in healthcare costs in the amount of 1.137 trillion US dollars and would help save 30.2 million life years over 30 years (Dixon et al., 2023, p. e2341408).

To understand the principles of prescription and use of pharmacotherapy in the primary and secondary prevention of CVD, we consider it appropriate to focus on the cardiovascular continuum proposed in 1991 by American scientists V. Dzau and E. Braunwald (Dzau & Braunwald, 1991, pp. 1244–1263), who hypothesised the development of atherosclerosis from the moment of birth a person. CVD risk factors such as hypertension, dyslipidemia, diabetes, obesity, smoking and stress lead to structural changes in blood vessels and the development of atherosclerosis and left ventricular hypertrophy. The main manifestations of atherosclerosis are CHD, which is complicated by coronary thrombosis and myocardial infarction. If appropriate prevention is not carried out for such patients, heart rhythm disturbances occur over time, remodelling and dilatation of the left ventricle occurs, which subsequently leads to chronic heart failure (CHF) and the final stage of CVD. Incidentally, if there are interventions from the side of primary or secondary or tertiary prevention in any period of the development of CVD and the removal of modified risk factors, its final stage may be delayed in time (Dzau & Braunwald, 1991, pp. 1244–1263).

Thus, the model of providing pharmaceutical care to patients with CHD with comorbid conditions should take into account the cardiovascular continuum and pharmacotherapy at various stages of disease progression.

Therefore, we consider it appropriate to take into account the frequency of prescribing certain pharmacological groups of medicines to patients with CHD and comorbid conditions (hypertensive disease, type II diabetes and metabolic syndrome). Thus, according to the data of the observational study EUROASPIRE V (De Smedt et al., 2020, pp. 7–13; Kotseva et al., 2020), in which participated 27 European countries (2017-2018), including Ukraine (n = 8261 patients), it was found out that 97% of patients took statins, therapy with antihypertensive effect (beta-blockers – 37.9%, ACE inhibitors – 51.7%, ARBs – 27.2%, BCC – 32%, diuretics – 35.6%, other medicines – 4.4%), 79.2% – antidiabetic medicines, 14.5% – insulins. 42.2% of patients involved in this study took one antihypertensive medicine, 34% – 2 medicines, 17.7% – 3 medicines, 6.1% – more than 4 antihypertensive medicines. At the same time, 67.6% of patients take 5 or more medicines used for CVD (De Smedt et al., 2020, pp. 7–13; Kotseva et al., 2020); anxiety and depression are observed in 43.8% (De Smedt et al., 2019), which indicates polypharmacy in the use of pharmacotherapy of CHD with comorbid conditions. According to the conclusions of this study, it is emphasised that the impact on risk factors (high blood pressure, dyslipidemia, obesity, diabetes, stress, depression) on the part of medical employees is insufficient and the standards of providing medical care need to be improved (Kotseva et al., 2020; Ferrannini et al., 2020, pp. 726–733). The expediency of involving pharmacists in interprofessional interaction as part of multidisciplinary teams will improve the quality of a personalised approach, prevent possible medicines interactions taking into account their metabolic profile and reduce the risks of adverse effects by checking medicines compatibility, which will lead to patient compliance with pharmacotherapy.

The analysis of the latest ESC and AHA clinical recommendations makes it possible to determine the pharmacological groups of medicines used in hypertension, CHD, heart failure (HF), atrial fibrillation (AF) and used for secondary and tertiary prevention of CHD and comorbid conditions (Table 1) (Joglar et al., 2023; Marx et al., 2023; Mancia et al., 2023; McDonagh et al., 2023, pp. 3627–3639). According to the AHA/ESC clinical recommendations for hypertension and left ventricular hypertrophy (Mancia et al., 2023), loop and non-loop diuretics, β-blockers, angiotensin-converting enzyme inhibitors (ACE inhibitors) or angiotensin II receptor blockers (ARBs), mineralocorticoid receptor antagonists (MRAs), calcium channels blockers (BCC) and their fixed combinations (BCC/ARB, BCC/iACE, iACE/BCC/diuretic, ARB/BCC/diuretic, iACE/diuretic, ARB/diuretic, iACE/beta-blocker, acetylsalicylic acid (ASA)/ b-blocker) are applied.

**Table 1.** The use of pharmacological groups of medicines in coronary heart disease in terms of the cardiovascular continuum (Joglar et al., 2023; Mancia et al., 2023; Marx et al., 2023; McDonagh et al., 2023, pp. 3627–3639).

	AH	CHD	HF	AF
Diuretics	+	+	+	–
B-blockers	+	+	+	+
ACE inhibitors/ARBs	+	+	+	+
MRAs	+	-	+	+
CCBs	+	+/-	-	-
Fixed combinations	+	+	+	+
Statins	-	+	+	+/-
ASA	-	+	+	-
Clopidogrel	-	+	+	-
Nitrates	-	+	-	-
Valsartan/Sacubitril	-	-	+	-
SGLT2 inhibitors	-	-	+	-
DOACs	-	+/-	+/-	+
Antiarrhythmic medicines	-	-	-	+/-

The use of pharmacological groups of medicines in coronary heart disease in terms of the cardiovascular continuum (Joglar et al., 2023; Mancia et al., 2023; Marx et al., 2023; McDonagh et al., 2023, pp. 3627–3639)

Pharmacotherapy of CHD (Marx et al., 2023; Virani et al., 2023) requires the prescription of statins, beta-blockers, ACE inhibitors/ARBs, nitrates (as required), ASA, clopidogrel or ticagrelor and their combinations with direct oral anticoagulants (DOACs) in individual doses. Pharmacotherapy of AF (Joglar et al., 2023) requires the use of DOACs, β-blockers to slow down the heart rate, additionally, the use of antiarrhythmic medicines is possible if necessary. HF is adjusted depending on the ejection fraction of the left ventricle (Mancia et al., 2023). Standard pharmacotherapy of HF includes beta-blockers, sacubitril/valsartan, ACE inhibitors/sartans, MRAs and it is advisable to consider sodium-glucose cotransporter-2 inhibitors (SGLT2 inhibitors) and diuretics (Maddox et al., 2024).

It should be noted that in cases of CHD with heart failure (HF) (Marx et al., 2023; McDonagh et al., 2023, pp. 3627–3639), fixed combinations such as aspirin/statin with ACE inhibitors/ARBs, calcium channel blockers and diuretics may be used. In type 2 diabetes, fixed combinations of metformin with SGLT2 inhibitors can be used to achieve target blood glucose levels. The use of fixed combinations is recommended for such patients to improve adherence to treatment.

The scientific publications and clinical recommendations on pharmacotherapy of patients with coronary heart disease and comorbid conditions analysed by us allow us to state that the pharmacotherapy of patients with CHD with comorbid conditions, based on the principles of evidence-based medicine, includes a sufficiently large number of medicines, which may lead to a number of problems in the future. We determined that such problems include polypharmacy, interaction of medicines in the prescribed pharmacotherapy and with food, possible side effects and side effects in fixed combinations making it impossible to determine the side effects for a specific medicines, the affordability of medicines and the presence in the list of the main medicines (Cabinet of Ministers of Ukraine, 2023) (list of the main medicines recommended by WHO) (WHO, n.d.), ease of use and dosage regime. In Ukraine, the problems of the physical availability of pharmaceuticals, especially in rural areas and near-front areas, the proper storage of pharmaceuticals (long-term power outages) and their disposal, and the lack of pharmaceutical personnel in pharmacies are added to a number of the listed issues.

**Figure 1.**
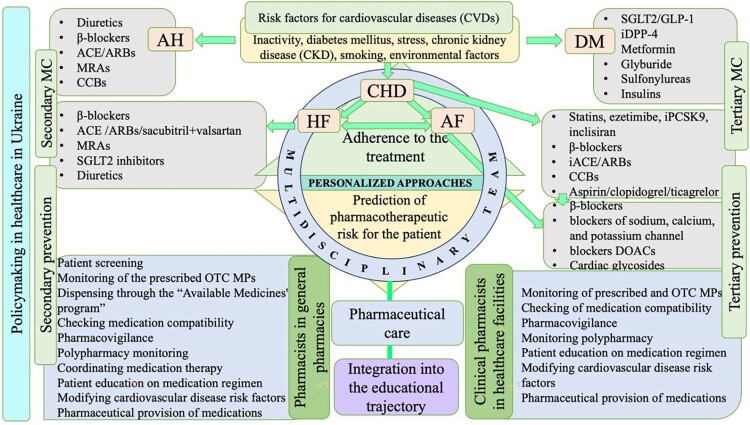
Development of a conceptual model of pharmaceutical care for patients with coronary heart disease with comorbid conditions (by the author). Note: MC – medical care; OTC MPs – over-the-counter medical products; AH – arterial hypertension.

Considering the issues we have examined regarding the development and reorientation of pharmaceutical care, the safety of using medications in cardiovascular diseases, and the involvement of pharmacists in interprofessional collaborations within the multidisciplinary teams, as well as the goal of alleviating the burden on the national healthcare system, we have justified and developed a conceptual model of pharmaceutical care for patients with CHD with comorbid conditions (such as diabetes mellitus, heart failure, atrial fibrillation, and chronic kidney disease (CKD)) (Figure 1).

The conceptual model of pharmaceutical care makes it possible to determine the place and role of pharmacists in the provision of pharmaceutical care in structure of medical care pharmacies by general pharmacists in the primary and secondary prevention of CHD with comorbid conditions and in hospital pharmacies by clinical pharmacists.

Thus, in the primary prevention of coronary heart disease with comorbid conditions in general pharmacies, in addition to placing information on the websites of pharmacy networks, it is advisable to use information brochures that are popular with patients (DASH diet, dietary nutrition for type II diabetes, etc.) due to the low socio-economic status of some segments of the population in wartime conditions; training on the use of mobile apps for a healthy lifestyle; monitoring of blood pressure (BP) and heart rate (HR); maintaining diaries of BP and HR, etc. (Bilousova, 2024b, pp. 42–52).

In cases of secondary and tertiary prevention of CHD with comorbid conditions, in addition to primary prevention measures, it is necessary to determine in secondary prevention all medicines that the patient is taking. Practical experience shows that doctors do not always have complete information about which medicines a patient can take in addition to prescribed pharmacotherapy, and the metabolic profile of medicines should be taken into account in order to prevent the occurrence of unwanted medicines interactions and side effects in future (Grześk et al., 2021, p. 8531).

We consider it expedient to identify another problem of the Ukrainian retail segment of the pharmaceutical market. The data of the Ukrainian monitoring companies indicate the trends of the market of dietary supplements to a sharp increase and decrease of the medicines market in monetary terms. At the same time, there is a decrease in prices due to the domestic exchange rate of the US dollar and a decrease in the pharmaceutical market in packages (Kirsanov, 2024). It can be safely assumed that a large number of Ukrainian patients take dietary supplements in addition to prescribed pharmacotherapy for CHD and comorbid conditions.

Therefore, after determining pharmacotherapeutic risks by pharmacists, it would be advisable to correct and coordinate pharmacotherapy with doctors. Such actions are possible over the phone or through the Ukrainian cloud-based eHealth programme (National Health Service of Ukraine, 2024), which is used to dispense drugs under the ‘Affordable Medicines' programme (Cabinet of Ministers of Ukraine, 2021) and currently does not allow doctors of various specialities to collect a complete list of medicines taken by a specific patient (National Health Service of Ukraine, 2024).

All of the mentioned above needs to be implemented in educational programmes up to the diploma level and in the process of continuous professional development (CPD) of special knowledge and development of competencies in the clinical pharmacology of medicines, safety of medicines use and improvement of communication skills. It should be noted that the set of proposed measures is partially implemented in educational measures for doctors and pharmacists in the CPD process through the Ukrainian ‘Association of Cardiovascular Care of Family Medicine' on the WebCardio.Org online-platform (WebCardio & org, n.d.).

Thus, the measures proposed by us in order to implement into the Ukrainian pharmaceutical practice the provision of pharmaceutical care to patients with coronary heart disease and comorbid conditions and the expansion of the roles of GPP require the adoption of certain political decisions in the national medical policy.

**Discussion.** Our analysis of Ukrainian regulatory and legal legislation highlights positive developments in the country, particularly in the pharmaceutical healthcare sector, in the context of implementing global best practices and European integration. For example, in the pre-war period, the first multidisciplinary teams, including pharmacists, were introduced to enhance infection control and prevent antibiotic resistance. This initiative demonstrated the benefits of interprofessional collaboration among all participants in the treatment process (Ministry of Health of Ukraine, 2024).

During the war in Ukraine, pharmacy institutions played a vital role in implementing vaccination and immunisation programmes for the population (Ministry of Health of Ukraine, 2024) However, the war has also exposed significant challenges, including a critical shortage of pharmaceutical personnel. This shortage is attributed to various factors, such as the displacement of pharmacists from occupied and front-line territories, insufficient social protection for pharmaceutical workers, and early burnout. Moreover, the monopolisation of the pharmacy business has shifted the focus of pharmaceutical services away from patient care towards maximising revenue through the sale of medicines. This includes promoting dietary supplements from internal pharmacy chain brands, driven by the structure of pharmacists’ salaries (Bilousova, 2024c, pp. 82–92; Corruption risks in the medical technology assessment procedure, 2023; Tkachenko, Zarichna et al., 2024, pp. 73–81). The discrepancies between updated Ukrainian legislation and its practical implementation during the war underscore the need for political will and enhanced oversight by state authorities over all participants in the medical process within the triad of ‘doctor-pharmacist-patient.' These challenges motivated us to scientifically substantiate the conceptual model of pharmaceutical care for patients with coronary heart disease with comorbid conditions.

In our recent empirical study (n = 101), we reliably confirmed low adherence to treatment in patients with coronary artery disease (CAD) with comorbid conditions who participated in the EUROASPIRE V observational study. Additionally, it was observed that these patients often discontinue pharmacotherapy upon experiencing an improvement in their well-being, largely due to the decline in socio-economic status associated with the ongoing war in Ukraine. Our findings also demonstrated that patients who received additional consultations from a pharmacist had significantly higher chances of improving their adherence to treatment (OR = 22.67), with a 22-fold increase in likelihood (Bilousova & Dolzhenko, 2024, pp. 6–8). Furthermore, we substantiated and summarised the functional roles of pharmacists in providing pharmaceutical care to CHD patients with comorbid conditions across primary (Bilousova, 2024d, pp. 42–52), secondary, and tertiary levels of medical care (Bilousova, 2024b, pp. 41–50). The results enabled us to generalise and adapt the concept of pharmaceutical care for CHD patients with comorbidities to contemporary conditions of healthcare delivery. These findings also align with other clinical studies, reinforcing the critical role of pharmacists in enhancing treatment adherence.

It is important to note that this study did not evaluation and sustainability of the conceptual model of pharmaceutical care for patients with CHD with comorbid conditions within the Ukrainian healthcare system. These aspects will be the focus of our future scientific research. However, we have synthesised global experiences in pharmaceutical policy and practice, while considering the unique features of Ukrainian legislation and the current practical implementation of the pharmaceutical component in medical care for CHD patients with comorbidities, particularly within the triad of ‘doctor-pharmacist-patient.'

The strengths of our study include a comprehensive approach to addressing the relevant research topic, utilising various research methods supported by a well-founded and reliable methodology. This study enabled the generalisation of international experience and highlighted value-oriented approaches to interdisciplinary collaboration within multidisciplinary teams. Additionally, a scientific foundation was established to address the unique features of Ukrainian pharmaceutical practice under conditions of limited resources and ongoing war. The results obtained can serve as a conceptual basis for testing and further practical implementation in Ukraine, taking into account European requirements.

The weaknesses of the study include the lack of empirical data and details regarding practical implementation, which were not the focus of our research but were considered in previous studies. The authors acknowledge that this study is limited in its assessment of the impact of the conceptual model of pharmaceutical care for patients with coronary artery disease and comorbid conditions on the healthcare budget, as well as in involving key stakeholders – doctors, pharmacists, and patients. These aspects will be the subject of future scientific research.

## Conclusions

1. Based on the results of the analysis of the above mentioned evidence base of the effectiveness of providing pharmaceutical care and the clinical recommendations of the AHA/ESC, the introduction of a conceptual model of pharmaceutical care for patients with coronary heart disease and comorbid conditions into pharmaceutical practice was developed and substantiated.
2. The place and role of pharmacists of general pharmacy institutions and healthcare institutions in terms of interprofessional collaboration in multidisciplinary teams is determined.
3. The expediency of involving pharmacists in interprofessional collaboration as part of multidisciplinary teams is substantiated, which will improve the quality of a personalised approach, prevent possible medicines interactions taking into account their metabolic profile, reduce the risks of side effects and lead to patient compliance with pharmacotherapy.
4. The proposed model of pharmaceutical care for patients with coronary heart disease and comorbid conditions takes into account the cardiovascular continuum and pharmacotherapy, which will lead to a decrease in disease progression, pharmacotherapeutic risks, reduce the burden on the healthcare system and guarantee the safety of medicines use.
5. Implementation of the conceptual model of pharmaceutical care for patients with ischemic heart disease and comorbid conditions in pharmaceutical practice requires revision of educational master’s training programmes and CPD programmes.

## Data Availability

All data generated and analysed are included in this research article.

## References

[CIT0002] Alshehri, A. A., Jalal, Z., Cheema, E., Haque, M. S., Jenkins, D., & Yahyouche, A. (2020). Impact of the pharmacist-led intervention on the control of medical cardiovascular risk factors for the primary prevention of cardiovascular disease in general practice: A systematic review and meta- analysis of randomized controlled trials. *British Journal of Clinical Pharmacology*, *86*(1), 29–38. 10.1111/bcp.14164 31777082 PMC6983518

[CIT0003] APhA. (n.d.). Clinical Guidelines. (USA). https://www.pharmacist.com/clinical-guidelines.

[CIT0004] Babar, Z.-U.-D. (2024). Building an effective medicines optimisation model: A health system approach. *International Journal of Clinical Pharmacy*, 1237–1242. 10.1007/s11096-024-01765-3 38896393

[CIT0005] Bilousova, N. (2024a). *Personalized approaches to the provision of pharmaceutical care: a modern request of Ukrainian society*. Management and marketing as part of modern economy, science, education, practice; 21 March 2024; Kharkiv, NPhaU p. 151–159. (Ukraine). https://mmf.nuph.edu.ua/wp-content/uploads/2024/03/zbirnik-konferencii-21.03.2024_10.04.pdf.

[CIT0006] Bilousova, N. (2024b). Justification for expanding the pharmacist’s role in the prevention of coronary heart disease at secondary and tertiary levels of medical care. *Modern Medicine, Pharmacy and Psychological Health*, *3*, 41–50. 10.32689/2663-0672-2024-3-7

[CIT0007] Bilousova, N. (2024c). Pharmaceutical care for patients with coronary heart disease: Status, problems and prospects. *Pharmaceutical Journal*, *4*, 82–92. 10.11603/2312-0967.2024.4.14991

[CIT0008] Bilousova, N. A. (2024d). Primary prevention of ischemic heart disease in the structure of pharmaceutical care: Functional fulfillment of pharmacist roles. *Pharmaceutical Journal*, *3*, 42–52. 10.11603/2312-0967.2024.3.14862

[CIT0009] Bilousova, N., & Dolzhenko, M. (2024). The influence of drug characteristics on adherence to treatment in patients with ischemic heart disease with comorbid conditions. In ZDMFU (Zaporizhia State Medical and Pharmaceutical University) (Ed.), *Zaporizhzhia pharmaceutical forum – 2024* (pp. 6–8). ZDMFU. (Ukraine). https://sites.google.com/view/zpf-2024/збирник-тез?authuser = 0.

[CIT0010] Bilousova, N. A., & Mykhalchuk, V. M. (2024). Analytical review of the influence of socio-economic factors on the state of pharmaceutical care for patients with cardiovascular diseases. *Polski Merkur Lek.*, *52*(3), 347–355. 10.36740/merkur202403112 39007474

[CIT0011] Cabinet of Ministers of Ukraine. (2021). *Some issues of reimbursement of medicines and medical products under the program of state guarantees of medical care of the population*. Appendix 1. List of reference countries., Resolution of the Cabinet of Ministers of Ukraine No. 854 dated 07/28/2021 No. 854. (Ukraine). https://zakon.rada.gov.ua/rada/show/854-2021-п#n146.

[CIT0012] Cabinet of Ministers of Ukraine. (2022). *On medical products, law of Ukraine No. 2469-IX*. Cabinet of Ministers of Ukraine. (Ukraine). https://zakon.rada.gov.ua/laws/show/2469-20#Text.

[CIT0013] Cabinet of Ministers of Ukraine. (2023). *On making changes to the national list of essential medicines*, Resolution of the Cabinet of Ministers of Ukraine No. 18. (Ukraine). https://zakon.rada.gov.ua/laws/show/18-2023-п#Text.

[CIT0014] Chronic Disease Management. (n.d.). Home. https://www.pharmacist.com/Practice/Patient-Care-Services/Chronic-Disease-Management.

[CIT0015] Content restricted - ESCP. (n.d.). ESCP. https://escpweb.org/membership/guidelines-and-tools-for-clinical-pharmacy-practice-education-and-research/.

[CIT0016] Corruption risks in the medical technology assessment procedure (Strategic analysis of corruption risks). (2023). National Agency for Corruption Prevention. https://nazk.gov.ua/wp-content/uploads/2023/06/doslidzhennya-OMT-2023.pdf.

[CIT0017] De Smedt D, De Backer T, Petrovic M, De Backer G, Wood D, Kotseva K, De Bacquer D. (2020). Chronic medication intake in patients with stable coronary heart disease across Europe: Evidence from daily clinical practice. Registry. Int J Cardiol, 300, p. 7-13. 10.1016/j.ijcard.2019.09.015. 31744720

[CIT0018] De Smedt D, Kotseva K, De Backer G, Wood D, Van Wilder L, De Bacquer D. (2019). EQ-5D in coronary patients: What are they suffering from? Results from the ESC EORP European survey of cardiovascular disease prevention and diabetes (EUROASPIRE IV) Registry. Qual Life Res, 29(4), p. 1037-1046. 10.1007/s11136-019-02334-2. 31741215

[CIT0019] Dixon, D. L., Johnston, K., Patterson, J., Marra, C. A., & Tsuyuki, R. T. (2023). Cost-Effectiveness of pharmacist prescribing for managing hypertension in the United States. *JAMA Netw Open*, *6*(11), e2341408. 10.1001/jamanetworkopen.2023.41408 37921763 PMC10625044

[CIT0020] Dzau, V., & Braunwald, E. (1991). Resolved and unresolved issues in the prevention and treatment of coronary artery disease: A workshop consensus statement. *Am Heart J. Flower*, *121*(4), 1244–1263. 10.1016/0002-8703(91)90694-d 2008853

[CIT0021] ESC Guidelines & Scientific Documents. (n.d.). European society of cardiology. https://www.escardio.org/Guidelines.

[CIT0022] Euro Commission. (2023). Resolution CM/Res (2020)3 on the implementation of pharmaceutical care for the benefit of patients and Health services: Resolution no. CM/Res (2020)3. https://rm.coe.int/09000016809cdf26.

[CIT0023] Ferrannini, G., De Bacquer, D., De Backer, G., Kotseva, K., Mellbin, L., Wood, D., & Rydén, L. (2020). Screening for glucose perturbations and risk factor management in dysglycemic patients with coronary artery disease – A persistent challenge in need of substantial improvement: A report from ESC EORP EUROASPIRE V. *Diabetes Care*, *43*(4), 726–733. 10.2337/dc19-2165 32079627

[CIT0024] FIP Library. (n.d.). *Home – FIP – International Pharmaceutical Federation home*. https://www.fip.org/publications?publicationCategory = 51.

[CIT0025] Grześk, G., Rogowicz, D., Wołowiec, Ł, Ratajczak, A., Gilewski, W., Chudzińska, M., Sinkiewicz, A., & Banach, J. (2021). The clinical significance of drug–food interactions of direct oral anticoagulants. *International Journal of Molecular Sciences*, *22*(16), 8531. 10.3390/ijms22168531 34445237 PMC8395160

[CIT0026] Guidelines and Statements. (n.d.). *Professional.heart.org*. https://professional.heart.org/en/guidelines-and-statements.

[CIT0027] Henman, M. C., Ravera, S., & Lery, F. X. (2024). Council of Europe resolution on the implementation of pharmaceutical care – A step forward in enhancing the appropriate Use of medicines and patient-centred care. *Healthcare*, *12*(2), 232. 10.3390/healthcare12020232 38255119 PMC10815874

[CIT0028] International Pharmaceutical Federation. (2019). *Beating non-communicable diseases in the community – The contribution on pharmacists*. The Hague: International Pharmaceutical Federation, p.127. https://www.fip.org/files/content/publications/2019/beating-ncds-in-the-community-the-contribution-of-pharmacists.pdf.

[CIT0029] International Pharmaceutical Federation. (2022). *Cardiovascular diseases: A handbook for pharmacists*. The Hague: International Pharmaceutical Federation, p. 24. https://www.fip.org/file/5252.

[CIT0030] International Pharmaceutical Federation (FIP). (2023). *Economic sustainability and pharmacy: A commentary article*. The Hague: International Pharmaceutical Federation, p. 21. https://www.fip.org/file/5870.

[CIT0031] Joglar, J. A., Chung, M. K., Armbruster, A. L., Benjamin, E. J., Chyou, J. Y., Cronin, E. M., Deswal, A., Eckhardt, L. L., Goldberger, Z. D., Gopinathannair, R., Gorenek, B., Hess, P. L., Hlatky, M., Hogan, G., Ibeh, C., Indik, J. H., Kido, K., Kusumoto, F., Link, M. S., … Van Wagoner, D. R. (2023). 2023 ACC/AHA/ACCP/HRS Guideline for the diagnosis and management of atrial fibrillation: A report of the American College of Cardiology/American Heart Association Joint Committee on Clinical Practice Guidelines. Circulation. 10.1161/cir.0000000000001193. PMC11095842 38033089

[CIT0032] Kharkiv Institute of Social Research – Public organization. (2024). Healthcare system in Ukraine: a test of resilience in times of war, p. 25. (Ukraine.). https://khisr.kharkov.ua/en/healthcare-system-in-ukraine-a-test-of-resilience-in-times-of-war/.

[CIT0033] Kirsanov, D. (2024). Pharmacy sales according to the results of the first half of 2024. Pharmacy online, 1449/1450 (28/29). (Ukraine). https://www.apteka.ua/article/698730.

[CIT0034] Kotseva, K., De Backer, G., De Bacquer, D., Rydén, L., Hoes, A., Grobbee, D., Maggioni, A., Marques-Vidal, P., Jennings, C., Abreu, A., Aguiar, C., Badariene, J., Bruthans, J., Cifkova, R., Davletov, K., Dilic, M., Dolzhenko, M., Gaita, D., Gotcheva, N., … Wood, D. (2020). Primary prevention efforts are poorly developed in people at high cardiovascular risk: A report from the European Society of Cardiology EURObservational research program EUROASPIRE V survey in 16 European countries. *European Journal of Preventive Cardiology*, (4), 370–379. 10.1177/2047487320908698 33966079

[CIT0035] Kovalenko, V. M., Lutai, M. I., Sirenko, Y. M., & Sychov, O. S. (2023). *Cardiovascular diseases. Classification, standards of diagnosis and treatment*. Kyiv. Chetverta khvylia, p. 84. (Ukraine). https://cardiohub.org.ua/wp-content/uploads/2024/02/CC3_2023.pdf.

[CIT0036] Levytska, O. R. (2020). Descriptive design of quality management of medical help for patients with acute cerebrovascular accidents in terms of pharmacotherapy. *Pharmaceutical Review*, *4*(56), 81–88. https://ojs.tdmu.edu.ua/index.php/pharm-chas/article/view/11645.

[CIT0037] List of “All documents” / Legislation of Ukraine. (n.d.). *Official web portal of the Parliament of Ukraine*. https://zakon.rada.gov.ua/laws.

[CIT0038] Maddox, T. M., Januzzi, J. L., Allen, L. A., Breathett, K., Brouse, S., Butler, J., Davis, L. L., Fonarow, G. C., Ibrahim, N. E., Lindenfeld, J., Masoudi, F. A., Motiwala, S. R., Oliveros, E., Walsh, M. N., Wasserman, A., Yancy, C. W., & Youmans, Q. R. (2024). 2024 ACC expert consensus decision pathway for treatment of heart failure with reduced ejection fraction. J Am Coll Cardiol. Birch. 10.1016/j.jacc.2023.12.024. 38466244

[CIT0039] Malanchuk, N. V., Demchuk, M. B., & Hroshovyi, T. A. (2022). Study of the range of antihypertensive medicines and analysis of their economic availability under the conditions of implementation of the government program “affordable medicines”. *Pharmaceutical Review*, *4*(64), 26–34. 10.11603/2312-0967.2022.3.13540

[CIT0040] Mancia, G., Kreutz, R., Brunström, M., Burnier, M., Grassi, G., Januszewicz, A., Muiesan, M. L., Tsioufis, K., Agabiti-Rosei, E., Algharably, E. A., Azizi, M., Benetos, A., Borghi, C., Hitij, J. B., Cifkova, R., Coca, A., Cornelissen, V., Cruickshank, K., Cunha, P. G., … Kjeldsen, S. E. (2023). 2023 ESH guidelines for the management of arterial hypertension The Task Force for the management of arterial hypertension of the European Society of Hypertension Endorsed by the European Renal Association (ERA) and the International Society of Hypertension (ISH). J Hypertens; Publish Ahead of Print. 10.1097/hjh.0000000000003480. 37345492

[CIT0041] Marx, N., Federici, M., Schütt, K., Müller-Wieland, D., Ajjan, R. A., Antunes, M. J., Christodorescu, R. M., Crawford, C., Di Angelantonio, E., Eliasson, B., Espinola-Klein, C., Fauchier, L., Halle, M., Herrington, W. G., Kautzky-Willer, A., Lambrinou, E., Lesiak, M., Lettino, M., McGuire, D. K., … Zeppenfeld, K. (2023). 2023 ESC guidelines for the management of cardiovascular disease in patients with diabetes. *European Heart Journal*, *44*(39), 4043–4140. 10.1093/eurheartj/ehad192 37622663

[CIT0042] McDonagh, T. A., Metra, M., Adamo, M., Gardner, R. S., Baumbach, A., Böhm, M., Burri, H., Butler, J., Čelutkienė, J., Chioncel, O., Cleland, J. G. F., Crespo-Leiro, M. G., Farmakis, D., Gilard, M., Heymans, S., Hoes, A. W., Jaarsma, T., Jankowska, E. A., Lainscak, M., … Skibelund, A. K. (2023). ESC scientific document group, 2023 focused update of the 2021 ESC guidelines for the diagnosis and treatment of acute and chronic heart failure: Developed by the task force for the diagnosis and treatment of acute and chronic heart failure of the European Society of Cardiology (ESC) With the special contribution of the heart failure association (HFA) of the ESC. *European Heart Journal*, *44*(37), 3627–3639. 10.1093/eurheartj/ehad195 37622666

[CIT0044] Ministry of Health of Ukraine. (2021). On approval of the Unified clinical protocol of primary, secondary (specialized) and tertiary (highly specialized) medical care “Stable coronary heart disease]. Ministry of Health of Ukraine. (Ukraine). https://www.dec.gov.ua/wp-content/uploads/2021/12/2021_2857_nakaz_stabihs.pdf.

[CIT0045] Ministry of Health of Ukraine. (2021). Unified clinical protocol of primary, secondary (specialized) and tertiary (highly specialized) medical care “Stable coronary heart disease”. MOZ Ukrainy. (Ukraine). https://www.dec.gov.ua/wp-content/uploads/2021/12/2021_2857_ykpmd_stabihs.pdf.

[CIT0046] Ministry of Health of Ukraine. (2022). On the approval of pharmacist protocols, Order of the Ministry of Health of Ukraine No. 7 (Ukraine). https://zakon.rada.gov.ua/rada/show/v0007282-22#Text.

[CIT0043] Ministry of Health of Ukraine. (2024, July 18). On amendments to the Order of the Ministry of Health of Ukraine No. 1254. Order of the Ministry of Health of Ukraine No. 1351. (Ukraine). https://zakon.rada.gov.ua/laws/show/z1183-24#.

[CIT0047] National Health Service of Ukraine. (2024). Electronic prescription for medicinal products: repayment details. Dynamics of the number of released packages according to ATC classification. (Ukraine). https://edata.e-health.gov.ua/e-data/dashboard/reimb-manufacturer-details.

[CIT0048] On the organization of infection prevention and infection control in healthcare institutions and institutions/facilities providing social services/social protection of the population (2023) Order of the Ministry of Health of Ukraine No. 1614 (Ukraine). https://zakon.rada.gov.ua/laws/show/z1318-21#Text

[CIT0049] Östbring, M. J., Eriksson, T., Petersson, G., & Hellström, L. (2021). Effects of a pharmaceutical care intervention on clinical outcomes and patient adherence in coronary heart disease: The MIMeRiC randomized controlled trial. *BMC Cardiovascular Disorders*, *21*(1), 367. 10.1186/s12872-021-02178-0 34334142 PMC8327441

[CIT0050] Practice Guidelines Resources | American Diabetes Association. (n.d.). Diabetes Professionals | American Diabetes Association. https://professional.diabetes.org/standards-of-care/practice-guidelines-resources

[CIT0051] State Expert Center of the Ministry of Health of Ukraine. (n.d.). State Expert Center of the Ministry of Health of Ukraine. https://www.dec.gov.ua/cat_mtd/galuzevi-standarti-ta-klinichni-nastanovi/

[CIT0052] Tkachenko, N., Pankevych, O., Mahanova, T., Hromovyk, B., Lesyk, R., & Lesyk, L. (2024). Human healthcare and Its pharmacy component from a safety point of view. *Pharmaceutical Review*, *12*(2), 64. 10.3390/pharmacy12020064 PMC11053725 38668090

[CIT0053] Tkachenko, N., Zarichna, T., Maganova, T., & Chervonenko, N. (2024). Modern approaches of pharmaceutical institutions to building long-term relationships with consumers of pharmaceutical products and improving pharmaceutical care. *Pharmaceutical Journal*, *4*, 73–81. 10.11603/2312-0967.2024.4.14990

[CIT0054] Virani SS, Newby LK, Arnold SV, Bittner V, Brewer LC, Demeter SH, Dixon DL, Fearon WF, Hess B, Johnson HM, Kazi DS, Kolte D, Kumbhani DJ, LoFaso J, Mahtta D, Mark DB, Minissian M, Navar AM, Patel AR, Piano MR, Rodriguez F, Talbot AW, Taqueti VR, Thomas RJ, van Diepen S, Wiggins B, Williams MS. (2023). 2023 AHA/ACC/ACCP/ASPC/NLA/PCNA guideline for the management of patients with chronic coronary disease: A report of the American Heart Association/American College of Cardiology Joint Committee on Clinical Practice Guidelines. Circulation. 10.1161/cir.0000000000001168. 37471501

[CIT0055] WebCardio.org. (n.d.). Electronic scientific and practical journal of cardiology. Postgraduate education: Cardiology. (Ukraine). https://www.youtube.com/@WebCardioOrg/videos.

[CIT0056] WHO. (n.d.). *EMLs around the world*. https://global.essentialmeds.org/dashboard/countries/2.

[CIT0057] World Health Organization. (1996). Good Pharmacy Practice (GPP) in community and hospital pharmacy settings, WHO/PHARM/DAP/96.1, p. 10. https://iris.who.int/bitstream/handle/10665/63097/WHO_PHARM_DAP_96.1.pdf?sequence = 1&isAllowed = y.

[CIT0058] World Health Organization (WHO). (2019). Medication safety in transitions of care. https://www.who.int/publications/i/item/WHO-UHC-SDS-2019.9.

[CIT0059] World Health Organization (WHO). (n. d.). Sustainable development goals. https://www.who.int/data/gho/data/themes/sustainable-development-goals.

[CIT0060] Yatskova, G. Y., Maksymovych, N. M., & Zaliska, O. N. (2019). Directions of optimization of information providing of pharmaceutical prophylaxis care for arterial hypertension. *Farmatsevtychnyi Zhurnal*, *1*(1), 31–42. 10.32352/0367-3057.1.19.03

[CIT0061] Zamorano, J. L. (2008). Heart rate management: A therapeutic goal throughout the cardiovascular continuum. *European Heart Journal Supplements*, *10*(Suppl F), 17–21. doi:10.1093/eurheartj/sun022

